# Injection Barrel/Nozzle/Mold-Cavity Scientific Real-Time Sensing and Molding Quality Monitoring for Different Polymer-Material Processes

**DOI:** 10.3390/s22134792

**Published:** 2022-06-24

**Authors:** Kai-Fu Liew, Hsin-Shu Peng, Po-Wei Huang, Wei-Jie Su

**Affiliations:** 1 Program of Mechanical and Aeronautical Engineering, Feng Chia University College of Engineering and Science, Taichung 407802, Taiwan; liew5927@mail.fcu.edu.tw (K.-F.L.); bowei8915@gmail.com (P.-W.H.); p0659828@mail.fcu.edu.tw (W.-J.S.); 2 Department of Mechanical and Computer Aided Engineering, Feng Chia University College of Engineering and Science, Taichung 407802, Taiwan

**Keywords:** foresight-work of scientific injection molding, melt pressure-sensors, viscosity index calculation, physicochemical properties of polymer, final-formed product quality

## Abstract

Scientific injection molding technologies involve the integration and collaboration of cyber-physical systems and smart manufacturing. In order to achieve adaptive process control and production optimization, injection molding systems with real-time sensing have gradually become the development- and application-trend of smart injection molding. At the same time, this technology is a highly non-linear process in which many factors affect the product quality during long-run fabrication processes. Therefore, in order to grasp changes in the characteristics of plastic materials and product quality monitoring, the injection process has become an important research topic. We installed sensors in the molding machine (injection barrel, nozzle, and mold-cavity) to collect the melting pressure and used different materials (semi-crystalline and amorphous polymer; the melting-fill-index (MFI) is unified to 14.5 ± 0.5 g/10 min) to explore the influences of melting pressure variation and its viscosity index on the quality characteristics of molded products. The experiment reveals that a combination of barrel, nozzle, and mold-cavity sensing on the melt-pressure trend-based injection process-control incorporated with viscosity index monitoring can confirm the weight and shrinkage variation of the injection product. At the same time, the pressure and viscosity index value measured and calculated during the melt-filling of two materials with similar MI resulted in significant variations in the amorphous polymer. This study showed the possibility of mastering and controlling the rheology (barrel position) and shrinkage properties of polymers and successful application in various product-quality monitoring platforms.

## 1. Introduction

**Figure 1:**
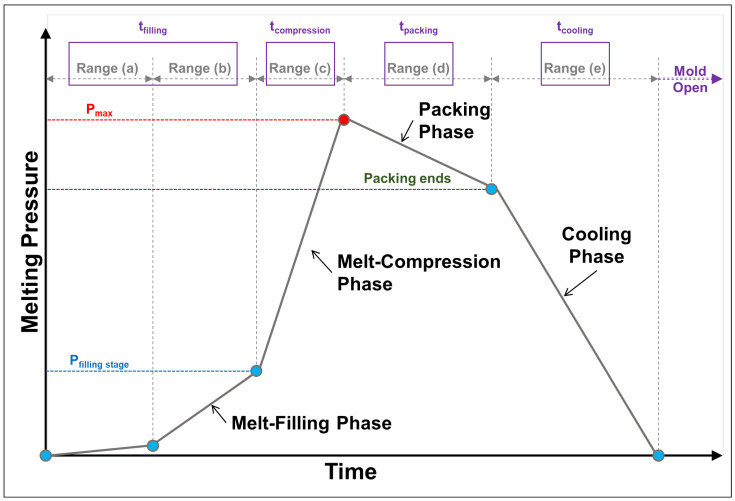
Trend chart of melting pressure for the basic injection process.

Scientific injection molding technologies involve the integration and collaboration of cyber-physical systems and smart manufacturing. In order to achieve adaptive process control and production optimization, the injection molding system with real-time sensing has gradually become the development and application trend of smart injection molding in the rubber and plastics industries [1,2,3,4]. Injection molding is a highly non-linear process in which many factors affect the product quality during long-run fabrication processes. To grasp changes in the characteristics of plastic materials and to improve product quality and machine productivity, information collected from real-time sensors during the injection molding process has become an important research topic. The P-V-T (pressure-specific volume-temperature) relationship is the basic characteristic of polymer materials and the specific volume of polymer materials changes with the molding temperature or pressure [5]. However, the change in process parameters and the effect on the properties of the molded material will make it difficult to predict the compressibility of plastic materials and product quality during injection molding. On the other hand, thermoplastic polymers currently represent a serious issue for various participants in the industry to manufacture irregular-shaped pieces requiring advanced mechanical properties; according to the formulation of polymers and microstructure (amorphous, semi-crystalline), a given thermoplastic can exhibit a wide range of end-use shrinkage properties [6,7,8]. In the case of polymer materials, the P-V-T relationship is very important because there is a strong relationship between pressure, volume, and temperature and the specific volume significantly affects the product weight. Using the relationship between pressure and temperature to maintain a specific volume can lead to a constant product weight [9,10]. However, the polymer injection molding process can be divided into seven stages: plasticization, clamping, injection, packing, cooling, mold-opening, and product ejection. Injection and packing are the stages that have been suggested to have the greatest effects on product quality [11,12,13,14]. A typical melting pressure-course profile is shown in Figure 1.

First, range (a) and range (b) represent the filling stage; range (a) occurs as the melt contacts the pressure sensor and gradually generates and reaches a stable-increase pressure trend; range (b) is the pressure trend when the melt enters the cavity. Range (c) is the value after the melt has completely filled cavity and enters the compression stage; the pressure value of this stage is the maximum value of the filling stage, and it indicates the end of filling and entry into the next stage (range (d)). However, if the pressure at the gate or sprue sufficiently high, the pressure compresses the melt. Thereafter, the melt within the cavity is maintained at an assigned pressure during the packing phase when additional plastic melt can be packed into the cavity to compensate for the plastic shrinkage caused by cooling to maintain a completely filled mold. This process continues until the gate is frozen, as marked at range (d). The final cooling phase comes afterward and continues to the end of the cycle (range (e)). It is during this phase that the melt gradually solidifies as the coolant that circulates within the cooling channels in the mold removes the heat. The cooling and solidification rates determine the speed of the pressure decrease. The melting pressure influences the quality of the finished molded product (shrinkage properties, dimensional stability, and mechanical behavior) [4,5,14,15,16,17,18,19,20]. In addition, quality indices can help to rapidly determine the quality of finished products without the need for any measuring equipment, a process that is feasible for quality control in actual injection molding operations. Although injection-molded finished products are not without defects, their quality has improved considerably because of the aforementioned advancements. The present study focused on PP and PS molding finished products with a thickness of 4 mm and investigated the melt-flow characteristics and its pressure-trend change. Additionally, the calculation formula (Equation (1), [1,2,5,21,22,23]) of a viscosity index was introduced to compare the effects of PP and PS under the same and different MI parameters. During injection molding, properties of the material can vary considerably due to the difference between semi-crystalline (PP) and amorphous (PS) polymer materials; at the same time, a higher melting pressure is generally required in order to accurately obtain samples’ weight/dimensional. Therefore, we sought to characterize the material properties during the injection molding process by formulating a VI (“VI” refers to the viscosity index, “scr−pos” refers to the screw position (“scr−pos 1” is when the melt contacts the pressure sensor, and the filling-start time (t); “ scr−pos 2” is when the melt reaches the V/P switch-over position and expresses the filling time-end (t)), and $P_{Melt}$ refers to the melting pressure). We used a pressure sensor to measure the melting pressure in order to calculate the *VI*. Pressure sensors are generally mounted in the injection barrel, nozzle, or mold-cavity.

(1) $$VI_{Injection}=\int_{t=scr-pos\;2}^{t=scr-pos\;1}P_{Melt}\left(t\right)dt$$

In this study, the changeover position was deemed a key factor in controlling the weight/dimensional accuracy (roundness) of samples, which meant that the pressure profile during the filling stage was more important than the pressure profile during the packing stage; at the same time, real-time measurement during an injection molding process can be used as a factor of the quality characteristics of the sample after forming to calculate the melt-viscosity index during the injection process. The molding material was selected based on the MFI of 14.5 ± 0.5 g/10 min, (i) polypropylene (PP; semi-crystalline polymer) and (ii) polystyrene (PS; amorphous polymer) were used as the research objects. Additionally, this study constructed a 4-mm thick and 100-mm diameter round-shaped sample and injection mold to observe melt-flow behavior. An experiment was also conducted to evaluate changes in the shrinkage properties of PP and PS by varying several parameter settings of the plasticizing and injection stages (plasticizing parameters are screw speed, back pressure, melt temperature; injection parameters are injection speed and V/P switch-over position).

## 2. Materials and Methods

### 2.1. Real-Time Sensing Design and Preparation

**Figure 2:**
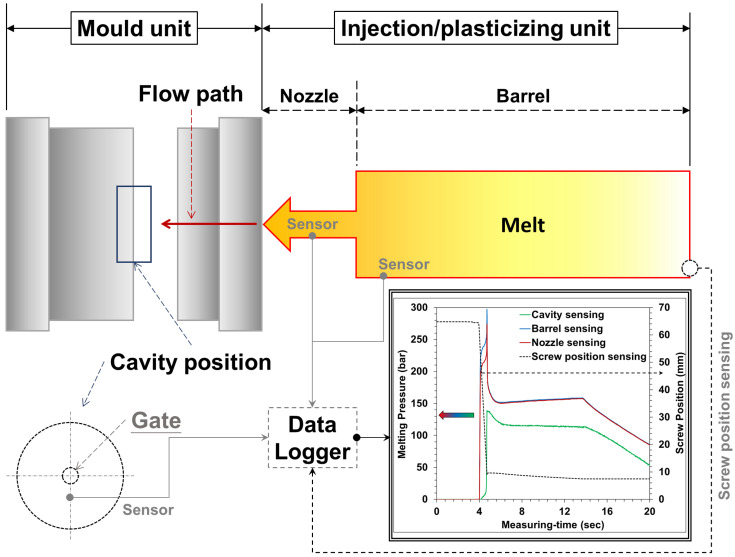
Schematic of different melt-pressure-sensor locations and their trend.

The sensors used in this study were based on the MFI of 14.5 ± 0.5 g/10 min and different molding materials (with or without crystallinity). Depending on its formulation and microstructure (amorphous, semi-crystalline), a given thermoplastic can exhibit a wide range of end-use shrinkage properties; the pressure-specific volume-temperature relationship is the basic characteristic of polymer materials and the specific volume of polymer materials changes with the molding temperature or pressure. Therefore, to grasp the shrinkage characteristics of the product, it is necessary to grasp the pressure change of the melt during the injection process and curing period. To investigate the different positions of melting pressure variations on the different molding materials, barrel, nozzle, and mold-cavity pressure sensors were set (Figure 2).

**Figure 3:**
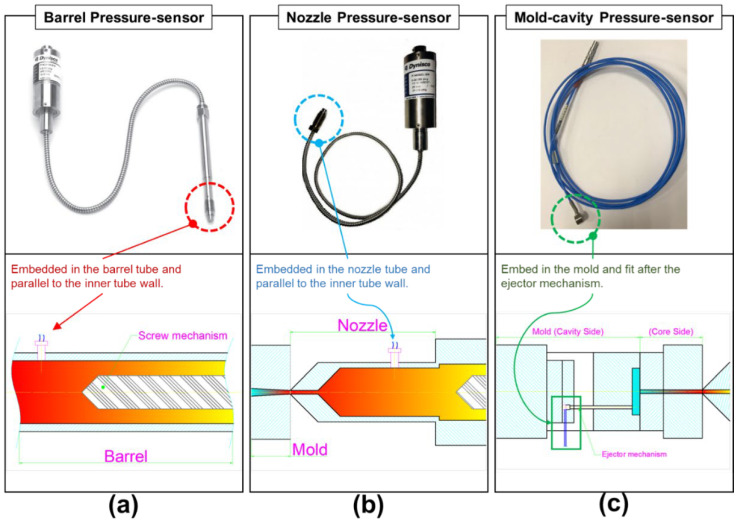
Schematic of sensors: (**a**) installed in the barrel-wall; (**b**) installed in the nozzle-wall; and (**c**) installed in the mold-cavity.

The barrel and nozzle pressure sensor (DYNISCO Agent; Taichung City, Xitun District, Taiwan; (i) PT4636-30M of model and installed in the position of the barrel; (ii) PT4656XL-30M of model and installed in the position of the nozzle) and the mold-cavity pressure sensor (FUTABA Corporation; Mobara, Japan) were installed as shown in Figure 3. This study conducted experiments on semi-crystalline (PP) and amorphous (PS) polymers. An injection molding process involving a scientific melt pressure measurement was employed, and the plasticizing and molding parameters, melting pressure, and viscosity index were analyzed and investigated. The sensor information is as follows:

- The sensor installed on the barrel and the nozzle acts directly on the diaphragm of the sensor through the pressure of a medium, causing the diaphragm to produce a slight displacement proportional to the pressure of the medium, thereby changing the resistance of the sensor and detecting it with its electronic circuit. It converts and outputs a corresponding pressure signal, which is also the value of the melting pressure required for this study (barrel and nozzle).
- As for the mold-cavity, it is a button-type sensor installed inside the mold-cavity. The pressure-sensing source comes from the ejector-pin in the mold. When the melt flows through the ejector-pin, a small displacement proportional to the medium pressure is also generated, which then causes the resistance of the sensor to change; the change detected by its electronic circuit, converted, and output as a corresponding pressure signal, which is also the melt pressure value in the mold-cavity required for this research.
- The non-linear error values of the above sensor are all 1.0 %F.S; and the available pressure range (maximum value) and material are (i) barrel and nozzle of 5~2100 bar and the material of Inconel-718 and (ii) mold-cavity of 5~800 bar and the material of SUS-630.

### 2.2. Molding Material and Its Equipment

**Figure 4:**
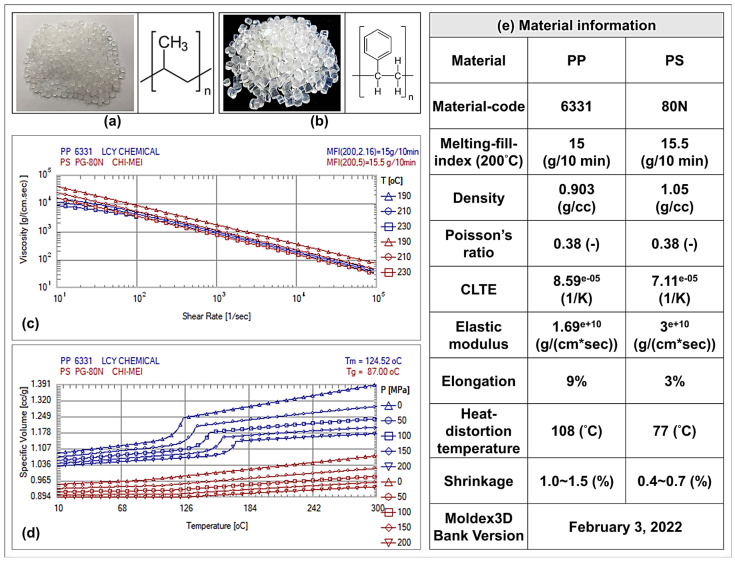
Schematic of experiment materials: (**a**) polypropylene (PP); (**b**) polystyrene (PS); (**c**) viscosity; (**d**) P-V-T relation; and (**e**) material information. (Source (**c**–**e**): Moldex3D Information Library).

**Figure 5:**
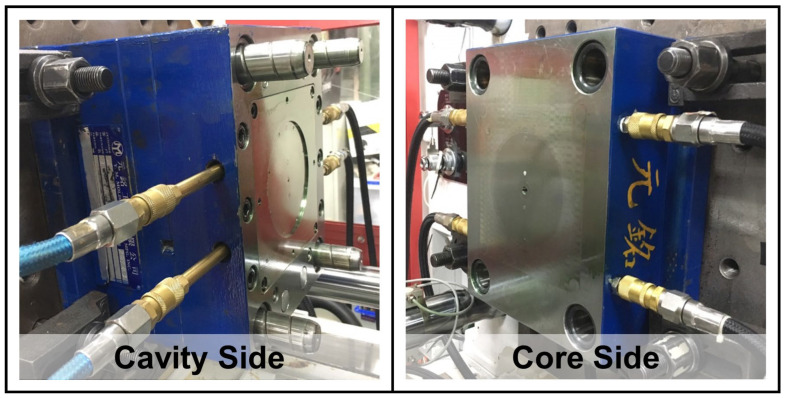
The mold of the round-shaped sample.

The molding materials used in this study were based on the melting-fill-index (MFI) of 14.5 ± 0.5 g/10 min, and (i) polypropylene (the material of semi-crystalline polymer; PP-6331 manufactured by LCY Group (Lee-Chang-Yung Chemical Co., Ltd.; Taipei City, Songshan District, Taiwan)) abbreviated here as PP and (ii) polystyrene (the material of amorphous polymer; PS-80N manufactured by CHIMEI Industry Co., Ltd. (Tainan City, Rende District, Taiwan)) abbreviated here as PS were used as the research objects (Figure 4). These two materials were selected because their materials are environmentally friendly, non-toxic, and low in material density. At the same time, in terms of semi-crystalline and amorphous materials, both are widely used materials. On the other hand, from Figure 4c–e, it can be seen that the viscosity variety and P-V-T characteristics of the materials are different due to the relationship of crystallinity (Ref: Moldex3D information library). Therefore, it is very important to observe the material flow through the sensor, and the influences of melting pressure and viscosity index on the quality characteristics of molded products during the injection process. The PP and PS pellets were dried for 4 h at 80 °C before use. As well as the melt fluidity and molding quality of PP and PS materials, round-shaped injection samples and molds were designed (Figure 5) with a sample size of 100 mm diameter and 4 mm thickness. In addition, the injection machine (basic model CLF-60TX) used in this study was jointly developed by the researchers and CLF (Chuan-Lih-Fa Machinery Works Co., Ltd.; Tainan City, Guanmiao District, Taiwan).

Table 1 shows the injection parameters; the temperature profile along the barrel was 210 °C (melt temperature), injection pressure was 170 bar, injection time was 1.5 s, injection speed was 70  mm/s and cooling time was 40 s, mold temperature was 60 °C, back pressure was 5 bar, screw speeds was 100 rpm, volume was fixed by a screw position of 45 mm, and V/P switch-over position was 10 mm (these parameters were defined as the standard/base molding conditions). The molding machine has a total mold clamp force of 60 tons; this machine had an ECO-STAR servo system of 22  kW (an accurate injection controller and procedure monitoring system were installed to ensure a stable molding process, plasticizing quality, and screw position control); a maximum injection rate of 115 cm^3^/s (injection-rotating single cylinder; screw/barrel-inner diameter is 30 mm and overall-length is 520 mm; injection quantity is the theoretical shot volume multiplied by the PS plastic coefficient of 0.91), and a maximum setting-value of injection pressure of 170 bar.

### 2.3. Characterizations

**Figure 6:**
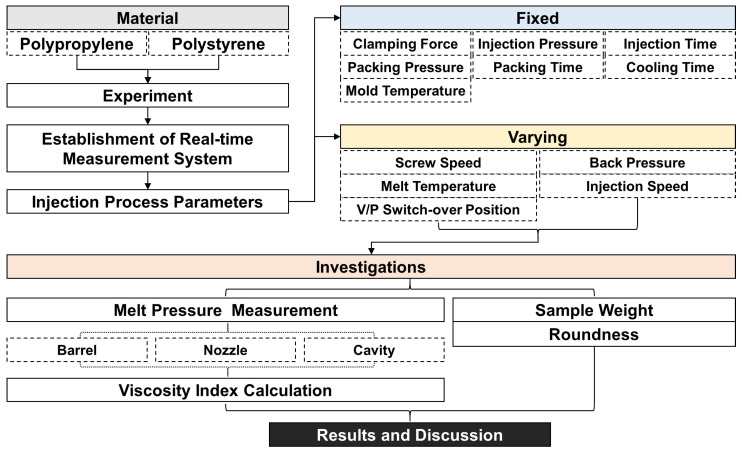
Flowchart of the DOE.

For the melting pressure measurement, 20 samples were tested under the same molding parameters. The average value of the last 15 test molded samples was used for analysis; details of the injection molding process used are shown in Figure 6. In addition to this, the effects of PP and PS on the melt pressure-peak, viscosity index, and molding quality variations of the injection course, as well as changes in the different molding parameters, i.e., (screw speed, back pressure, melt temperature, injection speed, and V/P switch-over position), and the correlated parameters of samples during the PP and PS injection molding process are listed in Table 2. 

In these experiments, back pressure was set to 5 bar, screw speed to 100 rpm, melt temperature to 210 °C, injection speed to 70 mm/s, and V/P switch-over position to 10 mm (these parameters were defined as the standard molding criteria).

**Figure 7:**
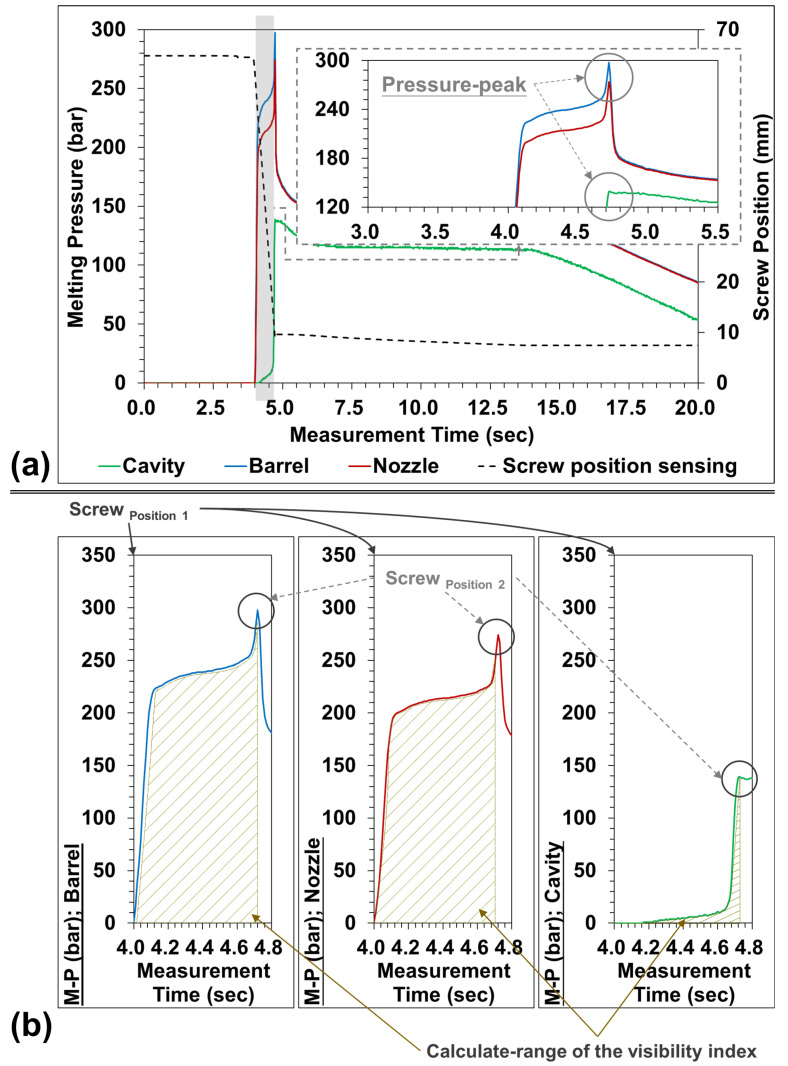
Melting pressure and viscosity index calculation: (**a**) melting pressure measurement; (**b**) calculation of the viscosity index.

**Figure 8:**
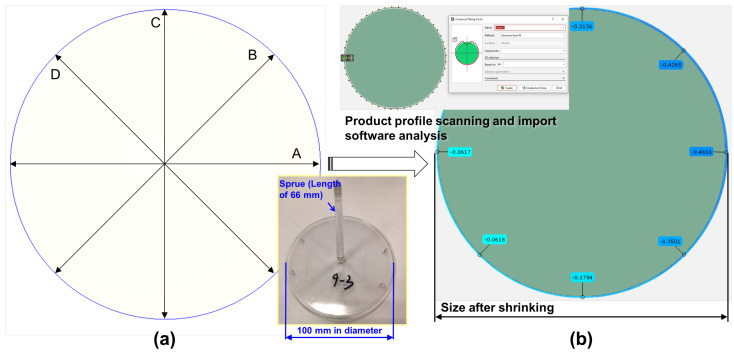
Schematic of the measurement of the finished product after shrinkage: (**a**) original size; (**b**) 3D−OMS measurement of the diameter of the finished product after shrinkage.

- In order to grasp the change of melting pressure during the injection molding process, the melt pressure was measured using the barrel, nozzle, and mold-cavity pressure sensor, and the pressure peak was measured at maximum pressure (Figure 7).
- The black-dotted line in Figure 7a is the original screw position sensor (SPS) of the injection machine. Through the SPS (scr−pos 1 to scr−pos 2) and the trend of the matching melt pressure, and combined with the Equation (1), the integral (*VI*) under the pressure and time is calculated (Figure 7b).

- The shrinkage part of the finished product was measured using a 3D optical measurement system (3D-OMS; ATOS Core 185 manufactured by Road-Ahead-Technologies Consult-ant Corp.; Taichung City, Daya District, Taiwan); the measurement range is 185 mm × 140 mm, the working distance is 250 mm, the charge-coupled device pixel is 12 million, and the measurement accuracy ±5%. The actual molding characteristics of the 3D-OMS will be used to measure the roundness (shrinkage characteristics) of the molded product and correlate it with the viscosity index. We carried out the radial measurement on a circular-part, which is divided into 8-point straight-line measurements, and Equation (2) was used to calculate the roundness variations (Figure 8).

(2) $$\Delta =R_{max}-R_{min}$$

## 3. Results

### 3.1. Effects of Material Variation and Melt-Fluidity Properties

1. Melting Pressure and Its Relevance to Viscosity Index

**Figure 9:**
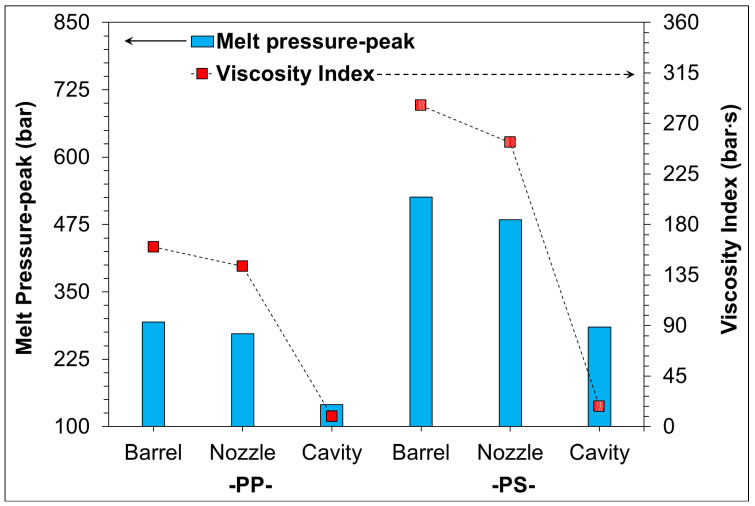
Trend-chart of melting pressure-peak and viscosity index at different positions for PP and PS.

During the injection molding process, the process parameters (plasticization and injection) of plastic polymers will be changed, thus affecting melt-fluidity and its molding characteristics. Therefore, in this study, the melting pressure variation of two different plastic materials from melt plasticization to injection was recorded and calculated by means of pressure sensors, and the melt pressure-peak and viscosity index were calculated. Figure 9 shows the trend of the melt pressure-peak and viscosity index measured at different sensing locations (barrel, nozzle, and cavity) for PP and PS materials under standard DOE from plasticizing to injection filling. The results showed that, although the melt indices of the plastic materials were similar and the molding parameters remained unchanged, the measured pressure-peak and viscosity indices of the two materials still varied significantly; among them, the PS material responded more to the larger changes in melt pressure-peaks and viscosity indices. At the same time, the P-V-T characteristics of both indicate that the molecular chain of PP material is sparser than that of PS under the influence of pressure and temperature (refer to Figure 4c,d), and when the melt is filled into the mold-cavity, the melting pressure in the mold-cavity can be established only when the melt is filled to the full capacity of the cavity. It can be thus observed that when molding PS materials, the melt flow resistance requires a higher molding pressure to push the plastic materials, making the melt pressure-peak and viscosity index of the PS greater than that of PP materials. Through the literature [1,2], it can be found that by installing the sensor at the barrel position and by sensing the pressure trend change of the melt, the pressure trends obtained are the same as at the nozzle position. Moreover, by installing the sensor at the barrel position, it is also possible to grasp the changes in the plasticizing and injection conditions of the material.

2. Melt-fluidity and Its Effect of Quality Characteristics

**Figure 10:**
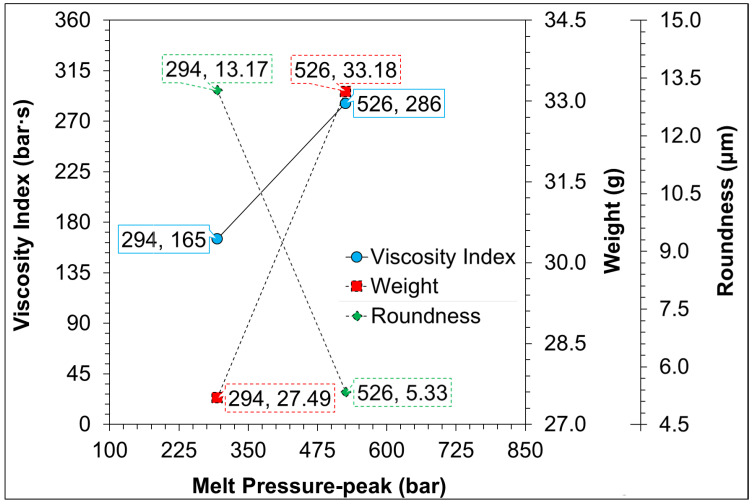
Effect of different materials on melt pressure-peak (*x*-axis) and viscosity index (continuous-line) on the barrel and its sample weight/roundness (short-dash-line/chain-line) (*y*-axis); the melt pressure peak is 294 bar for PP and 526 bar for PS.

Conditions of injection molding are often adjusted to achieve better sample quality, and changes in DOE directly affect the melt filling and flow characteristics, molding pressure, and shrinkage characteristics, resulting in changes in the quality characteristics of the sample, such as weight, appearance, and size. Therefore, this stage measures the melting pressure and calculates the viscosity index, and observes the product quality of PP and PS. Figure 10 indicates the comparison between PP and PS in a single (standard/base) condition using the melt pressure-peak measured by the injection barrel and comparing the viscosity index, weight of the finished product after molding, and shrinkage characteristics (roundness). The results show that the melt pressure-peak of PP and PS materials are 294 bar and 526 bar, respectively, and the calculated and measured data can be obtained on the Y-axis corresponding to the viscosity and P-V-T characteristics curve provided in the materials property data. From the trend, we found that the viscosity index of the melt, the weight of the finished product, and the change in the roundness are all positively correlated. This shows the pressure information collected through the injection barrels’ sensor is an excellent reference value and is useful to grasp and monitor the injection conditions for the pressure change during the melt filling and the quality characteristics of the sample after molding. Therefore, follow-up research will use the pressure sensor at the position of the barrel as the basis for comparing different molding parameters with their melt pressure-peak, viscosity index, and quality characteristics.

### 3.2. DOE and Its Correlation-Analysis of Quality Characteristics

**Figure 11:**
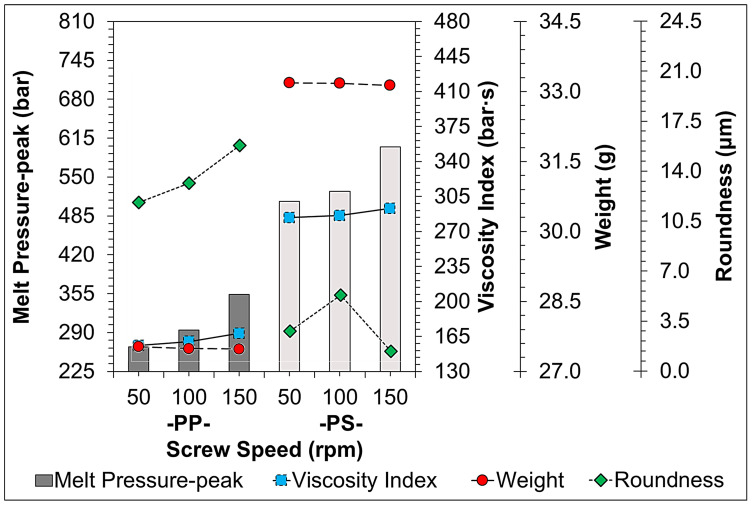
Trend-diagram of the effect of screw speed on melt pressure-peak (bar-graph), calculated viscosity index (continuous-line), and its sample weight (chain-line)/roundness (dotted-line).

**Figure 12:**
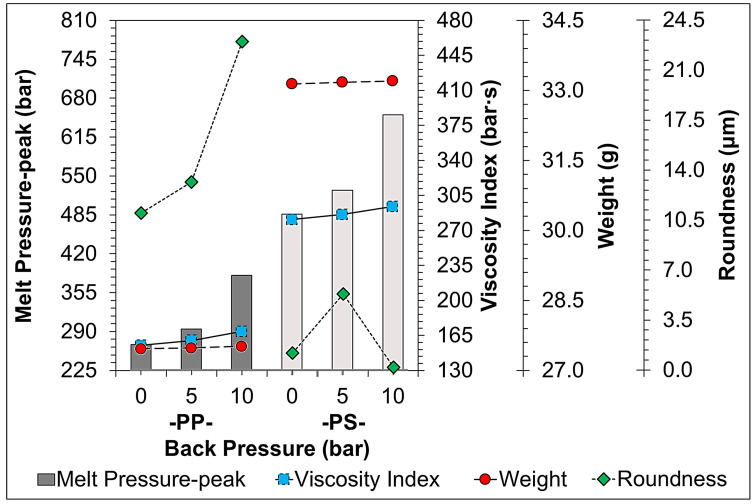
Trend-diagram of the effect of back pressure on melt pressure-peak (bar-graph), calculated viscosity index (continuous-line), and its sample weight (chain-line)/roundness (dotted-line).

**Figure 13:**
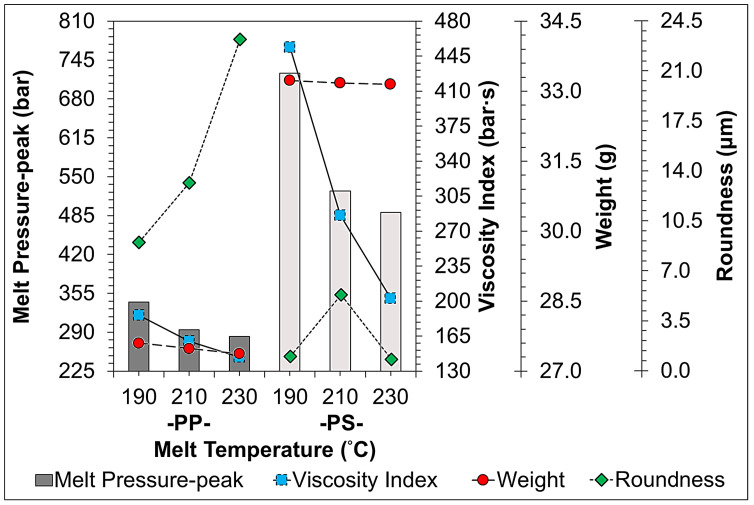
Trend-diagram of the effect of melt temperature on melt pressure-peak (bar-graph), calculated viscosity index (continuous-line), and its sample weight (chain-line)/roundness (dotted-line).

**Figure 14:**
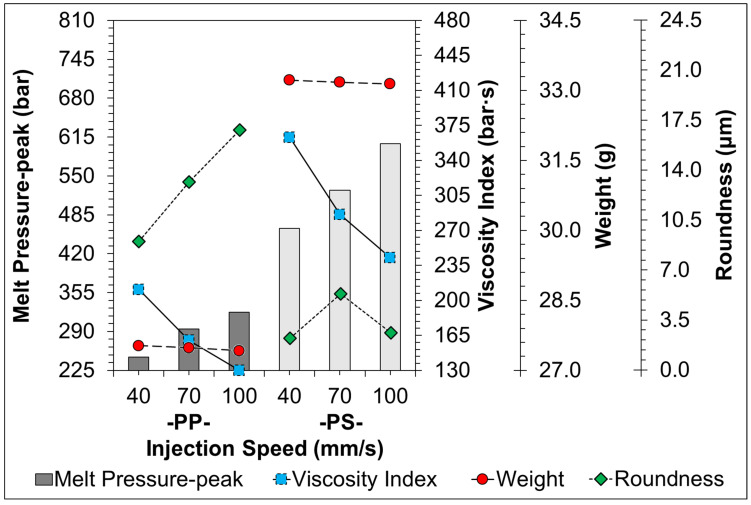
Trend-diagram of the effect of injection speed on melt pressure-peak (bar-graph), calculated viscosity index (continuous-line), and its sample weight (chain-line)/roundness (dotted-line).

**Figure 15:**
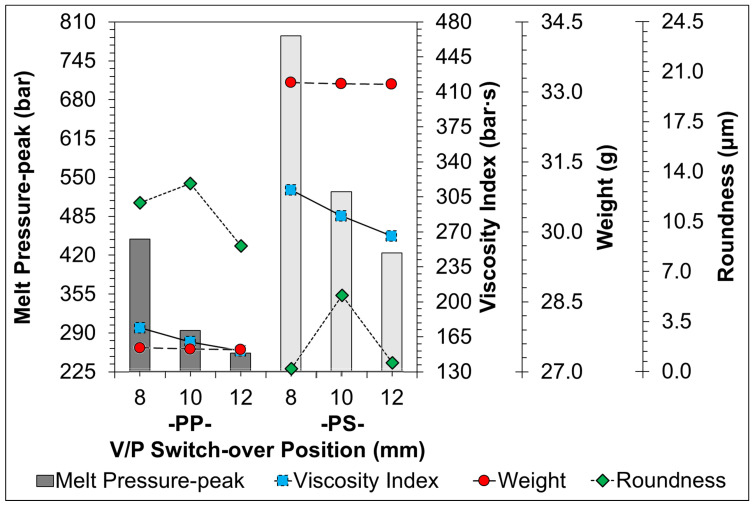
Trend-diagram of the effect of V/P switch-over position on melt pressure-peak (bar-graph), calculated viscosity index (continuous-line), and its sample weight (chain-line)/roundness (dotted-line).

Figure 11, Figure 12, Figure 13, Figure 14 and Figure 15 indicate the comparison of the melt pressure-peak of semi-crystalline (PP) and amorphous polymers (PS) under different molding conditions with respect to the viscosity index and sample weight/roundness; at the same time, the injection-molded samples, product thickness, gate location, and gate type may cause a high degree of molecular orientation in the flow direction due to the high shear effect during the resin-filling stage. This molecular orientation effect also causes anisotropy or asymmetry in the overall shrinkage of the sample, i.e., the plastic shrinks inconsistently in the direction of flow and the direction of vertical movement. At this time and in general, the screw speed and back pressure play a key role in the plasticization quality of plastic materials during the plasticization stage, and the trend of the effect on the weight of the sample and viscosity index after the plasticization stage by the pressure measurement (melt pressure-peak) at the front end of the barrel during the injection process is illustrated in Figure 11 (Description 1) and Figure 12 (Description 2).

1. Screw Speed Variation

It can be clearly observed from Figure 11 that as the screw speed increases, the melt pressure-peak and viscosity index correspondingly increase, which also reflects the need for a larger molding capacity, and the faster the screw speed, the faster the plastic material is transported, the shorter the storage time, and the higher the pressure of the injection barrel. On the other hand, it can also be seen that as the screw speed increases, the weight tends to decrease slightly. At the same time, among the screw speed parameters affecting the shear rate and molecular chain of the molten material, an increase in screw speed and shear rate increases the destructiveness of the material molecular chains, resulting in a significant increase in the roundness of the PP where the semi-crystalline has a greater effect on the contractility.

2. Back Pressure Variation

Among the two main plasticizing parameters affecting the stability and flow ability of the molten material, an increase in screw speed and back pressure increases the destructiveness of the material molecular chains. Proper back pressure adjustment can improve the mixing and uniformity of the melt during plasticization. Figure 12 shows the trend of the change of melt pressure-peak during injection when adjusting back pressure conditions in response to the viscosity index and the weight/roundness of the sample. The results indicate that the higher back pressure increases the shear of the screw, increases the density of the material in the barrel, therefore, significantly increasing the melt pressure-peak, while the weight of the sample also increases. An increase in back pressure increases the destructiveness of the material molecular chains and its damaged and sparser molecular chains can lead to the inability to support shrinkage forces during melt-cooling and curing, resulting in a significant increase in the roundness of the sample, where the back pressure has a greater effect on the destructiveness of the molecular chains and shrinkage forces of PP.

3. Melt Temperature Variation

Figure 13 shows the trend of the change of melt pressure-peak during injection when adjusting melt temperature conditions in response to the viscosity index and the weight/roundness of the sample. As the melt temperature increases, the viscosity of the plastic decreases and the flowability of the melt increases, resulting in a decrease in the melt pressure-peak trend and viscosity index. Furthermore, as the melt temperature increased, the PS showed a greater change in the melt pressure-peak and viscosity index, indicating that the melt temperature had a more significant effect on the flow characteristics of PS; since PP is a semi-crystalline material, the P-V-T characteristics show that the melt temperature has a more significant variation on the specific volume, resulting in a greater change in the sample weight. It can be clearly seen that the viscosity index of both PP and PS plastics tends to significantly decrease with the increase in melt temperature; the PS material is more obviously affected by the melt temperature. In the comparison of the roundness, the roundness and viscosity index of PP plastic showed opposite trends, and the roundness was more obviously affected by the melt temperature.

4. Injection Speed Variation

Figure 14 shows the trend of the change of melt pressure-peak during injection when adjusting injection speed conditions in response to the viscosity index and the weight/roundness of the sample. In the experiment to adjust the injection speed, it can be clearly seen that as the injection speed increases, the melt flow resistance decreases, causing the melt pressure-peak and viscosity index to relatively decrease. In addition, due to the faster injection speed, there is no trend observed of sample weight increase due to the influence of the reaction force inside the cavity; at the same time, the effect of changing the injection speed can be clearly seen in that the viscosity index of both PP and PS plastics tends to significantly decrease with the increase in injection speed. In the comparison of the roundness, the roundness and viscosity index of PP plastic showed opposite trends; due to the faster injection speed, it is easy to form a mold-cavity full of melt and form sparse molecular chains. Therefore, although the influence on the viscosity index is negatively correlated, the reaction injection speed should not be used as the basis for improving shrinkage.

5. V/P Switch-over Position Variation

Figure 15 shows the trend of the change of melt pressure-peak during injection when adjusting V/P switch-over position conditions in response to the viscosity index and the weight/roundness of the sample. From the figure, we can clearly see that the density of the material obtained in the cavity is lower when the packing pressure is switched early, and the weight of the finished product is also lower. On the contrary, late switching of the packing pressure will result in a more molten resin filling of the mold-cavity, increasing the density of the material and the weight of the finished product. Whether it is PP or PS material, the melt pressure-peak is obviously affected by the V/P switch-over position, which shows that this condition plays an important role in the adjustment of melt pressure-peak and viscosity index and of the sample weight. In the injection molding process, when the high-temperature plastic material is injected into the mold-cavity and then cooled, the specific volume of the materials continues to decrease during the cooling process due to the polymer material properties. This cooling process causes a reduction in volume, which is the shrinkage of the plastic material. Crystalline plastics have a larger shrinkage value because crystallization occurs during the cooling process and molecular chains interact with each other to form crystalline regions, resulting in further volume reduction, resulting in a larger shrinkage value for crystalline plastics.

## 4. Conclusions

This study conducted experiments on semi-crystalline (PP) and amorphous (PS) polymers. An injection molding process involving a scientific melting pressure measurement was employed, and the plasticizing and molding parameters, melt pressure-peak, viscosity index, and molding quality (sample weight, roundness) were analyzed and investigated. The following points should be noted:

1. Through the use of a real-time pressure measurement system, we were able to successfully grasp the pressure history and the trend changes in the viscosity index of the melt from the plasticization to the injection process (injection barrel to the mold-cavity).
2. A 3D-OMS analysis which integrates the actual molding characteristics of the injection machine by sensing information was successfully used to measure the roundness (shrinkage characteristics) of the sample and compare it with the melt pressure-peak and viscosity index.
3. Real-time melt pressure-peak (viscosity index) information collected from sensors during the injection molding process can be used as a characteristic value of adaptive process-control for scientific injection molding.

## Figures and Tables

**Table 1:** Configuration of the molding parameters for the molding of PP and PS samples.

Factors (Set Value) Maintained Constant		Controllable Factors			
Clamping Force (ton)	45	Screw Speed (rpm)	50	100	150
Mold Temperature (°C)	60	Back Pressure (bar)	0	5	10
Cooling Time (s)	40	Melt Temperature (°C)	190	210	230
Injection Pressure (bar)	170	Injection Speed (mm/s)	40	70	100
Injection Time (s)	1.5	V/P Switch-over Position (mm)	8	10	12
Packing Time (s)	10				
Packing Pressure (bar)	10				

**Table 2:** Random order of the treatments for molding of the PP and PS samples.

Exp.	Screw Speed (rpm)	Back Pressure (Bar)	Melt Temperature (°C)	Injection Speed (mm/s)	V/P Switch-Over Position (mm)
1	50	5	210	50	10
2	100	5	210	50	10
3	150	5	210	50	10
4	100	0	210	50	10
5	100	5	210	50	10
6	100	10	210	50	10
7	100	5	190	50	10
8	100	5	210	50	10
9	100	5	230	50	10
10	100	5	210	40	10
11	100	5	210	70	10
12	100	5	210	100	10
13	100	5	210	50	8
14	100	5	210	50	10
15	100	5	210	50	12

## Data Availability

The data presented in this study are available upon request from the corresponding author.
